# Wood Sent to Jail

**Published:** 1905-02

**Authors:** 


### WOOD SENT TO JAIL.

Dr. N. News Wood, Chicago, Ill., of Christian Hospital notoriety, was sentenced to ten days in jail for contempt of court, December 30, and in addition he was fined $100 and the "hospital" $250, for violation of an injunction restraining them from using the name of Dr. John B. Murphy in connection with the establishment.

